# Hospital emergency department visits made by developmentally disabled adolescents with oral complications

**DOI:** 10.3389/froh.2022.955584

**Published:** 2022-08-15

**Authors:** Kathryn A. Atchison, Vinodh Bhoopathi, Christine R. Wells

**Affiliations:** ^1^Section of Public & Population Health, School of Dentistry, University of California, Los Angeles, Los Angeles, CA, United States; ^2^Statistical Methods and Data Analytics, Office of Advanced Research Computing, University of California, Los Angeles, Los Angeles, CA, United States

**Keywords:** emergency department use, Medical Home, developmentally disabled children, adolescent oral health, Andersen's Behavioral Model of Health Services use, National Survey of Children's Health (NSCH), dental caries, children with special health care needs (CSHCN)

## Abstract

**Purpose:**

We used Andersen's Behavioral Model in a cross-sectional study to determine the factors associated with utilization of the emergency department (ED), controlling for whether an adolescent has a developmental disability (DD) and one or more oral complications (toothaches, decayed teeth, bleeding gums, eating or swallowing problems).

**Methods:**

Data from the 2016–2019 National Survey of Children's Health (NSCH) was used for this secondary data analysis study. We used frequencies and percentages to describe the sample characteristics. Chi-square tests were used for bivariate analyses. Multivariable logistic regression modeling was conducted to predict ED visits by adolescents aged 10–17 controlling for predisposing, enabling, and need variables.

**Results:**

The sample consisted of 68,942 adolescents who were primarily male, non-Hispanic White, and born in the U.S. Parents reported that 69% of the adolescents had neither a DD nor an oral complication; 10% had no DD but experienced one or more oral complication; 16% had a DD but no oral complication; and 5% had both DDs and one or more oral complication. Adolescents with both a DD and an oral complication reported the highest level of ED visits at 33%, compared to 14% of adolescents with neither DD nor oral complication. Regression analysis showed that adolescents with a DD and oral complication (OR: 2.0, 95% CI: 1.64–2.54, *p* < 0.0001), and those with DDs but no oral complications (OR: 1.45, 95% CI: 1.25–1.68, *p* < 0.0001) were at higher odds of having an ED visit compared to those with neither a DD nor an oral complication. Not having a Medical Home increased the likelihood of ED visits by 14% (*p* = 0.02). Those with private insurance (OR: 0.63, 95% CI: 0.53–0.75, *p* < 0.0001) and those from a family where the highest level of education was some college and above (OR: 0.85, 95% CI: 0.73–0.98, *p* = 0.03) were less likely than their counterparts to have had an ED visit.

**Conclusion:**

Adolescents with DDs and oral complications utilize ED visits more frequently than those with neither DDs nor oral complications. Integrating the dental and medical health systems and incorporating concepts of a Patient-Centered Medical Home could improve overall health care and reduce ED visits for adolescents.

## Introduction

Emergency Department (ED) use for non-traumatic dental care problems has been reported as a common and growing problem in the U.S. [1, 2]. Between 2000 and 2010, the number of dental visits to the ED doubled, and the proportion of ED visits as a percent of total dental visits increased [1]. Allareddy et al. [3] reported that among the ED visits, people 21 years of age and younger sought care for problems with dental caries, pulp and periapical diseases, diseases of the gingiva and periodontium, abscesses, and cellulitis. Studies of the care received by both adults and children within the ED confirm that definitive care for oral health problems is not provided. More appropriate care could better be provided within the dental office [3, 4]. Frequent users of the ED include people with Medicaid coverage, private insurance, the uninsured, and children with special healthcare needs (CSHCN) [5, 6].

CSHCN are defined as children with “chronic physical, developmental, behavioral, or emotional conditions who require health and related services of a type or amount beyond that required by children generally” [7]. While CSHCN are a heterogeneous group, poorer health status and various unmet needs (e.g., dental care, specialty care, prescription medicine) are common [5]. A combination of financial barriers (e.g., limited income to pay health care costs), practice-level barriers (e.g., couldn't get an appointment), and system-level barriers (e.g., couldn't get a referral) contribute to poor health outcomes and unmet needs. Lewis [8] reported that dental care was the most common unmet need and was second only to medications in the frequency of need. Cost of dental care was the most frequently reported reason for the lack of care receipt, particularly non-preventive dental care, followed by lack of insurance.

Caregivers of children with functional limitations report a variety of challenges to parenting, including having to stop or reduce their working hours, spend numerous hours providing or coordinating care for their CSHCN, experiencing financial difficulties associated with the child's condition, and are less likely to get the children to preventive dental care [9, 10]. Care coordination and the ease of provider referrals were two commonly reported needs by parents of youth with disabilities.

The Patient-Centered Medical Home (PCMH or Medical Home) is considered the standard for health care delivery among CSHCNs and their families [11]. Medical Home is a health care delivery model where primary care services are accessible, family-centered, continuous, comprehensive, coordinated, compassionate, and culturally effective [11]. Not having a Medical Home has been negatively associated with receiving dental care services and unmet dental care needs among CSHCNs [12, 13]. The Medical Home is designed to provide a patient-centered orientation, comprehensive, team-based care over the range of preventive, acute care, chronic care; coordinated care; access to care, including after-hours; and a systems-based approach to quality and safety [14].

Developmental disabilities (DDs), a category within CSHCN, represent a group of conditions that result from an “impairment in physical, learning, language, or behavior areas” that begin during the developmental period and may lead to a variety of functional impacts [15]. According to parental reports in the 2009 to 2017 National Health Interview Surveys, about one in six (17%) children aged 3–17 are diagnosed with a DD, and that number grew from 16.2% in the 2009–2011 survey to 17.8% in the 2015–2017 survey [16].

Adolescence is a critical time in which children develop positive and negative behaviors that will continue throughout the course of ones' life. Positive behaviors include making healthy food choices and amounts and good oral hygiene, and they can set the adolescent on a pathway for good health behaviors. Negative behaviors, such as smoking, vaping, alcohol use, or early sexual activity, lead to future health problems, including sexually transmitted diseases and chronic diseases [17]. The American Academy of Pediatrics indicates that children with DDs may have significant physical and cognitive issues, and those from low-income families are at high dental care risks [18]. National-level data (2015–2017) shows that adolescents aged 12–17 years have the highest prevalence of DDs at 21.1% in 2015–2017 compared to other children [16]. The other prevalent condition in adolescents of ages 12–19 is dental caries (tooth decay/cavities), with almost 57% of them having experienced it [19]. This age group also has the highest untreated dental caries prevalence (16.6%) compared to other age groups, indicating a greater increased unmet dental need. As described above, children with DDs are more likely to visit the ED compared to peers without DD. However, it is unclear whether oral complications increase the frequency of ED visits among adolescents with DDs or if other system-level factors contribute to high ED use. Therefore, using nationally representative data, our study aims to determine the factors associated with the utilization of the ED, controlling for whether or not the adolescent has a DD and/or an oral complication.

## Methods

In this cross-sectional analysis, the factors under consideration linked the predisposing, enabling, and need variables associated with the Andersen's Behavioral Model of Health Services [20] use with the health system factors that make up the PCMH.

### Data source

We used the data from the National Survey of Children's Health (NSCH) waves 2016, 2017, 2018, and 2019, sponsored by the Maternal and Child Health Bureau of the Health Resources and Services Administration. Briefly, the data were collected by the United States Census Bureau using a two-stage paper survey instrument in conjunction with a single-stage web-based survey instrument. During data collection, a screener questionnaire was used to identify households with non-institutionalized children ages 0–17 years and included a battery of questions to identify CSHCN. One child was randomly selected from each eligible household, and that child was the subject of a more detailed topical questionnaire completed by the parent or caregiver. It covers the 50 states and the District of Columbia. There were 131,774 children and adolescents ages 0–17 years in 2016 through 2019 NSCH. We analyzed only the subpopulation of adolescents aged 10 to 17 (*n* = 68,942) for these analyses.

### Variables

#### Dependent variable

The dependent variable is the parent's response to the question, “During the past 12 months, how many times did this child visit a hospital or emergency room?,” categorized as no visits or one or more visits.

#### Independent variables

The primary independent variable represents a combination of the adolescent's status regarding a DD and his/her oral health status, where four groups were created. We combined the two-parent reports that the child currently has one or more DD with the report of four oral complications. DDs included ASD (autism spectrum disorder), ADHD (attention deficit hyperactivity disorder), blindness, cerebral palsy, hearing loss/deafness, learning disability, intellectual disability, seizures in the past 12 months, stuttering or stammering in the past 12 months, or any other developmental delay [16]. It should be noted that “any other developmental delay” option was not assessed in 2019 NSCH. The parent's report on oral complications included whether or not the child experienced frequent or chronic difficulty with any of the following items in the past 12 months: tooth decay, toothaches, bleeding gums, or problems while eating or swallowing due to a health condition. Combining the two variables, we categorized subjects into four adolescent groups: (a) With a DD and one or more oral complications, (b) With a DD but no oral complication, (c) With No DD but having one or more oral complications, and (d) Neither a DD nor an oral complication.

#### Pre-disposing variables

Predisposing variables are the individual's social and demographic characteristics that may influence a person's decision to use a health service such as a dental office or an emergency department [20]. The predisposing variables that may affect the decision to utilize the ED considered in the study were: the adolescent's gender (male/female), race/ethnicity (Hispanic/non-Hispanic White/non-Hispanic Black/other), language spoken in the household (Spanish/English/other), the highest level of education in the household (less than high school/high school or GED/some college or technical school/college degree and above), and whether the child was born in the U.S. Age was not considered for the regression, as all participants were adolescents within a narrow age group (aged 10–17).

#### Enabling variables

Enabling variables are those conditions that describe the family's resources available to access services utilization [20]. There are four variables considered enabling variables in the dataset. First, the family's federal poverty level (FPL) (0–99% FPL; 100–199% FPL; 200–400% FPL; more than 400% FPL), is a measure of income issued annually by the Department of Health and Human Services. Those below 100% FPL are usually considered at the lowest end of the socioeconomic status. The price of health care to the family, determined by the individual's health insurance status (public only; private only; private and public; insurance not specified; not insured) was used as the second enabling variable. The third enabling variable was “having access to a Medical Home.” NSCH includes the following five components to measure the presence of a Medical Home: Personal doctor or nurse, the usual source for sick care, family-centered care, problems getting needed referrals, and effective care coordination when required. The final enabling variable included was whether the adolescent had a dental visit in the past 12 months representing an oral health enabling variable.

#### Health-related hardships and need

Need variables can include both individual-level factors and health-related conditions of the environment, for example, the community spending on services and a shortage of professionals available to provide care [20]. Need factors can also differentiate between the individual or parent's perceived need for health care and the evaluated need, such as an objective oral health measurement. Parents were asked to provide a “Yes” or a “No” response to each of four individual level oral health conditions listed: “During the past 12 months, has this child had frequent or chronic difficulty with any of the following”: toothaches, decayed teeth, bleeding gums, and eating or swallowing because of a health condition which was then summed to four categories (No oral complications, 1, 2, or 3 or more oral complications). In addition, parents were asked whether they had the health insurance coverage available when needed (Always, Usually, Sometimes, Never) and whether they were frustrated at getting the health services required for their child (Always, Usually, Sometimes, Never).

### Statistical analyses

Analyses were conducted only in the subpopulation of interest, adolescents aged 10–17 years. To account for the complex survey design of NSCH, we used survey weights to calculate all estimates. The appropriate primary sampling unit and strata variables were also included to correct the variance estimates for the complex survey design. All data were weighted to be representative of the U.S. population of non-institutionalized adolescents aged 10–17 years. Weighted percentages were derived to understand the demographic characteristics, the prevalence of DDs and oral complications, and other predisposing, enabling, and need variables related to the study sample. The sample for any analysis may be less than the total sample due to listwise deletion of missing data. Chi-square tests were conducted to determine the association between DD and oral complication status and utilization of ED visits in the past 12 months. Additionally, Chi-square tests determined the association between individual Medical Home components and ED visits. A logistic regression model for utilization of ED visits among adolescents in the past 12 months (One or more ED visits vs. no ED visit) was run, controlling for predisposing, enabling, and need variables. The statistical significance was set at *p* < 0.05. Analyses were conducted using Stata statistical software (17, StataCorp LLC, College Station, TX).

## Results

### Characteristics of the overall sample

The sample were primarily male (51%), non-Hispanic White (51%), from an English-speaking household (86%), and were born in the U.S. (95%) (Table 1). Parents reported that 69% of the adolescents had neither a DD nor an oral complication, 10% had no DD but experienced one or more oral complications, 16% had a DD, but no oral complications, and 5% had both DDs and one or more oral complication. The majority of the adolescents were covered by private health insurance (59%), 4.6% had both private and public insurance, 29% had only public health insurance, 0.5% the insurance type was not specified, and 7.2% were not insured. The sample represented reasonably well-educated families, with 69% of the adolescents' parents reported some college or technical education and above, and only 11.5% had less than high school education. Approximately 58% reported living at or above 200% FPL. Parents reported that 46% of the adolescents had a Medical Home, and 90% had a dental visit in the past 12 months. The adolescent's age, gender, household language, and whether the child was born in the U.S. were not significantly associated with the dependent variable and were excluded from regression analyses.

**Table 1:** Emergency room visits by adolescent predisposing, enabling, and healthcare needs.

**Characteristics**	**Overall (%)**	**No visits (%)**	**1 or more visit (%)**	***p*-value^a^**
**PRE-DISPOSING VARIABLES**				
**Gender**				
Male	51.1	83.4	16.6	0.25
Female	48.9	83.2	16.8	
**Race/ethnicity**				
Hispanic	25.5	81.7	18.3	**<0.0001**
Non-hispanic white	50.5	85.2	14.8	
Non-hispanic black	14.1	76.4	23.6	
Other	9.9	87.7	12.3	
**Household language**				
English	86	83	17	0.09
Spanish	11	82	18	
Other	3	88	12	
**Born in USA**				
Yes	95	83	17	0.60
No	5	83	17	
**Highest education level in household**				
Less than high school education	11.5	78.3	21.7	**<0.0001**
High school or graduate education diploma	19.9	79.4	20.6	
Some college or technical school	22.6	81.2	18.8	
College degree or higher	46.0	87.3	12.7	
**ENABLING VARIABLES**				
**Type of insurance coverage**				
Public only	28.8	75.9	24.1	**<0.0001**
Private only	58.9	87.3	12.7	
Private and public	4.6	75.1	24.9	
Insurance type not specified	0.5	74.5	25.5	
Not insured	7.2	86.0	14.0	
**Federal poverty level**				
0–99%	19.8	75.6	24.4	**<0.0001**
100–199%	21.9	81.7	16.3	
200–400%	27.2	85.1	14.9	
More than 400%	31.0	87.7	12.3	
**Presence of patient centered medical home**				
Yes	46.3	86.0	14.0	**<0.0001**
No	53.7	81.0	19.0	
**Dental visit in the past 12 months**				
Yes	90.0	83.4	16.6	0.14
No	10.0	82.9	17.1	
**HARDSHIPS FACED AND UNMET HEALTHCARE NEEDS (NEED VARIABLES)**				
**Developmental disabilities (DDs) and oral health complication status**				
No DD nor oral health complication	69.0	86.0	14.0	**<0.0001**
No DD but with oral complications	10.0	82.0	18.0	
With DDs but with no oral complication	16.0	78.0	22.0	
With both DDs and oral complications	5.0	67.0	33.0	
**Health insurance coverage when needed**				
Always	64.0	83.6	16.4	**0.008**
Usually	28.3	82.6	17.4	
Sometimes	6.3	80.6	19.4	
Never	1.3	85.0	15.0	
**Frustrated to get health services for child in past 12 months**				
Never	81.6	85.7	14.3	**<0.0001**
Sometimes	15.4	73.9	26.1	
Usually	1.9	67.8	32.2	
Always	1.1	65.8	34.2	
**Frequent or chronic difficulty in last 12 months due to decayed teeth**				
Yes	11.4	79.7	20.3	**<0.0001**
No	88.6	83.8	16.2	
**Frequent or chronic difficulty in last 12 months due to tooth aches**				
Yes	4.1	71.3	28.7	**<0.0001**
No	95.9	83.9	16.1	
**Frequent or chronic difficulty in last 12 months due to bleeding gums**				
Yes	2.7	72.3	27.7	**<0.0001**
No	97.3	83.7	16.3	
**Frequent or chronic difficulty in last 12 months with eating or swallowing due to health condition**				
Yes	1.4	52.9	47.1	**<0.0001**
No	98.6	83.7	16.3	
**Frequent or chronic difficulty in the last 12 months due to one or more oral health complications**				
No complications	85.0	84.5	15.5	**<0.0001**
1 complication	11.3	79.0	21.0	
2 complications	3.0	70.7	19.3	
3 or more complications	0.7	65.9	34.1	

a Chi-square tests comparing adolescents' ED visit vs. no ED visits by pre-disposing, enabling, and need factors.

Bold p-values indicate statistical significance at p < 0.05.

### Bivariate associations between adolescents with no ED visits vs. one or more ED visits by various pre-disposing, enabling, and need factors

Among the Predisposing variables, race/ethnicity and highest education level in household had statistically significant differences in their categories that reported one or more ED visits (*p* < 0.0001) (Table 1). Non-Hispanic Blacks and Hispanics were most likely to report one or more ED visits (23.6 and 18.3%, respectively). Families who reported higher ED visits included those with less than high school education (21.7%) and a High School or GED (20.6%). Of the Enabling variables, insurance type, income, and having access to PCMH had statistically significant differences in their categories that reported one or more ED visits (*p* < 0.0001). Families with Public only insurance (24.1%), Private and Public insurance (24.9%), and those with no specified insurance type (25.5%) reported higher ED visits than those with Private only or Not insured. Families who reported lower levels of income [FPL of 0–99% (24.4%) and FPL of 100–199% (18.3%) reported higher rates of ED visits than families with higher income levels. Families who reported the adolescent having access to a Medical Home (86%) were significantly less likely to have an ED visit, although the actual difference was small (14 vs. 19%). In contrast, having a dental visit in the past year was not associated with ED visits.

Adolescents with a DD were significantly more likely to have one or more ED visits (55%) than adolescents with no DD (32%), ignoring the impact of the oral complication (Table 1). The subset of adolescents with both a DD and one or more oral complications reported the highest level of ED visits at 33%, compared to 22% for adolescents with a DD but no oral complication, 18% for adolescents with no DD but who reported oral complications, and 14% for adolescents with no DD and no oral complication. Although parents reported both unmet dental and medical care, higher levels of unmet dental care (55%) were reported than unmet medical care (33%) (data not shown). Table 1 also presents the individual hardships, Developmental disabilities (DDs) and Oral health complication status (*p* < 0.0001), having health insurance coverage when needed (*p* = 0.008), frustrated to get health services for child in the past 12 months (*p* < 0.0001), frequent or chronic difficulty in last 12 months due to toothaches (*p* < 0.0001), decayed teeth (*p* < 0.0001), bleeding gums (*p* < 0.0001), with eating or swallowing due to health condition (*p* < 0.0001), and due to one or more oral health complications (*p* < 0.0001) parents reported by the adolescents' use of the ED.

Always “having health insurance coverage when needed” was associated with fewer ED visits (Never = 85%; compared to 83.6% for Always), as was “Never having frustration when trying to get health services for their child” (85.7%). Parents reported their child as having frequent or chronic difficulty with decayed teeth (11.4 %), toothaches (4.1%), bleeding gums (2.7%), and eating or swallowing due to a health condition (1.4%) that were each significantly associated with higher ED visits. The sum of the oral complications showed a strong positive association with ED visits (15.5% for one or more ED visits for No complications, 21.0% for one complication, 19.3% for two complications, and 34.1% for three or more complications).

### Association between medical home components and ED visits among adolescents

The bivariate association between the five components of the PCMH included in the NSCH survey, having a personal doctor or nurse, having a usual source of sick care, received family-centered care, having difficulty getting referrals, received care coordination, and reporting ED visits are presented in Table 2. Three of the five components were associated with the use of the ED. Not having a personal doctor/nurse or usual source of sick care showed no association. Within the sample, parents of 80% of adolescents reported the adolescent received some health care and answered follow-on questions about the PCMH. The 20% of the parents who reported they received no health care were not included in the PCMH questions. Of those receiving some health care, 68% of parents reported receiving family-centered care and 12% reported they did not receive family-centered care. Within the group who received some family-centered care, the adolescents required fewer ED visits (18% with one or more ED visits) than those who did not receive family-centered care (24%). With respect to access receiving referrals, among the 18% of the adolescents who required a doctor referral, parents reported it as “Not difficult” to obtain by the majority (14%) and 31% reported one or more ED visits, “Not possible to get” was reported by 0.2% of parents. Difficulty in getting needed referrals were strongly associated with ED visits (*p* < 0.0001), with 45% of their adolescents having one or more ED visits.

**Table 2:** Association between medical home component and reports of hospital emergency room visit.

**Medical home component**	**Overall (%)**	**No ED visits (%)**	**1 or more ED visit (%)**	***p*-value^a^**
**Personal doctor/nurse**				
Yes	72.0	83.0	17.0	0.55
No	28.0	84.0	16.0	
**Usual source of sick care**				
Yes	76.0	84.0	16.0	0.69
No	24.0	83.0	17.0	
**Received family centered care**				
Yes	68.0	83.0	18.0	**<0.0001**
No	12.0	76.0	24.0	
No health care	20.0%	91.0	9.0	
**Difficulty getting referrals**				
Did not need referrals	82.0	87.0	13.0	**<0.0001**
Not difficult	14.0	69.0	31.0	
Somewhat difficult	3.0	62.0	38.0	
Very difficult	0.8	42.0	58.0	
It was not possible to get referrals	0.2	55.0	45.0	
**Received care coordination**				
Did not need care coordination	47.0	90.0	10.0	**<0.0001**
Received needed care coordination	37.0	80.0	20.0	
Did not receive needed care coordination	16.0	72.0	28.0	

a Chi-square tests comparing adolescents with no ED visits vs. one or more ED visits by individual medical home components.

Bold p-values indicate statistical significance at p < 0.05.

### Multivariable logistic regression model determining factors associated with ED visits among adolescents

A logistic regression model was fit to understand the association between the primary independent variable (disability status and oral complications) and ED visits in the past 12 months, controlling for other predisposing, enabling, and need factors (Table 3). Compared to adolescents with neither a DD nor an oral complication, the odds of having ED visits increased by 45% if the child had a DD but no oral complication [Odds ratio (OR): 1.45, 95% CI:1.25–1.68, *p* < 0.0001)] and were twice as common if the adolescent had both a DD and one or more oral complication (OR:2.0, 95% CI:1.64–2.54, *p* < 0.0001). Having only an oral complication did not significantly increase the odds of an ED visit.

**Table 3:** Logistic regression model: characteristics of adolescents visiting hospital emergency department one or more times in the past 12 months.

	**Adjusted OR (95% CI)**	***p*-value**
**Developmental disabilities (DDs) and oral health complication status**		
With both DDs and one or more oral complications	2.0 (1.64–2.54)	**<0.0001**
With DDs but with no oral complication	1.45 (1.25–1.68)	**<0.0001**
No DDs but with one or more oral complications	1.18 (0.98–1.42)	0.07
Neither DD nor oral complication	Ref	
**Race/ethnicity**		
Other	1.1 (0.98–1.21)	0.12
Non-hispanic white	Ref	
**Type of insurance coverage**		
Private only	0.63 (0.53–0.75)	<**0.0001**
Private and public	1.03 (0.81–1.30)	0.79
Insurance type unspecified	0.94 (0.48–1.84)	0.86
Not insured	0.27 (0.14–0.52)	**<0.0001**
Public only	Ref	
**Insurance coverage when needed**		
Usually/sometimes/never	1.10 (0.99–1.23)	0.08
Always	Ref	
**Highest education level in household**		
Some college or technical school and above college degree	0.85 (0.73–0.98)	**0.03**
High school or graduate education diploma	Ref	
**Federal poverty level**		
More than 400%	0.85 (0.72–1.01)	0.07
200–400%	0.92 (0.78–1.09)	0.36
0–199%	Ref	
**Presence of medical home**		
No	1.14 (1.02–1.27)	**0.02**
Yes	Ref	
**Dental visit in the past 12 months**		
No	1.19 (0.98–1.46)	0.07
Yes	Ref	
**Frustration in getting care**		
Sometimes/Always/Usual	1.86 (1.62–2.13)	**<0.0001**
Never	Ref	

Bold p-values indicate statistical significance at p < 0.05.

Compared to having public only insurance, having private only insurance (OR: 0.63, 95% CI: 0.53–0.75, *p* < 0.0001) and being not insured (OR: 0.27, 95% CI: 0.14–0.52, *p* < 0.0001) were associated with lower odds of having an ED visit. Neither the combination of private and public nor having an unspecified insurance type was significantly associated with ED visits. Having some college or technical school and above college (OR: 0.85, 95% CI: 0.73–0.98, *p* < 0.03) was also protective. Experiencing frustration in getting care for their adolescent almost doubled the chance of an ED visit (OR: 1.86, 95% CI: 1.62–2.13, *p* < 0.0001). Likewise, not having a PCMH increased the likelihood of ED visits by 14% (OR: 1.14, 95% CI: 1.02–1.27, *p* = 0.02). There was no significant association between race/ethnicity, not having a dental visit in the past 12 months, insurance coverage when needed, or the parental FPL designation and ED visits.

### Characteristics of families having access to a medical home

Considering the potential importance of receiving good health care of having a PCMH, we compared the characteristics of the adolescents whom parents reported to have and did not have a Medical Home. Table 4 shows that adolescents having a Medical Home were more likely to not have a DD (47%); to be Non-White (55%); to have parents/care-givers with a college degree or higher (57%); to have Private only insurance (55%) and to have a family income >400% of the of the Federal Poverty Level (59.5%) (all *p* < 0.0001).

**Table 4:** Characteristics of adolescent disability status, demographic variables, and reported patient centered medical home.

**Characteristic**	**Has medical home %**	**No medical home %**	***p*-value^a^**
**Developmental disability status**			
Adolescents with DD	42.0	58.0	**<0.0001**
Adolescents without DDs	47.0	53.0	
**Race/ethnicity**			
Whites	38.0	62.0	**<0.0001**
Non-whites	55.0	45.0	
**Highest education level in household**			
Less than high school education	24.0	76.0	**<0.0001**
High school or graduate education diploma	36.0	64.0	
Some college or technical school	44.0	56.0	
College degree or higher	57.0	43.0	
**Type of insurance coverage**			
Private only	55.0	45.0	**<0.0001**
Private and public	38.0	62.0	
Insurance type unspecified	25.0	75.0	
Not insured	26.0	47.0	
Public only	36.0	64.0	
**Federal poverty level**			
0–199%	35.1	64.9%	**<0.0001**
200–400%	48.4	51.6	
More than 400%	59.5	40.5	

a Chi-square tests comparing adolescents with medical home vs. medical home.

Bold p-values indicate statistical significance at p < 0.05.

## Discussion

Emergency department visits associated with a dental condition almost doubled between 2000 and 2010 [2] and are acknowledged as an inefficient vehicle for providing comprehensive and effective dental care. This analysis of U.S. adolescents aged 10–17 confirmed that ~24% of adolescents have a DD, and these individuals had higher rates of ED visits than adolescents without a DD. The association between adolescents and ED visits was also higher for adolescents with a DD and one or more oral complications (such as decayed teeth, bleeding gums, or toothaches), not having a PCMH, and children whose parents report frustration in accessing health care for their adolescent. In contrast, having private only insurance or not being insured and having higher education were associated with having ED visits. While no information is available as to the reason that people with no insurance were less likely to have ED visits, it is possible that they were not users of the traditional health care system.

A study of children under 21 who accessed the ED for dental conditions occurred for diagnoses of dental caries, pulpal or periapical conditions, gingival/periodontal diseases, and abscesses [3]. CSHCN have been shown to have a high rate of dental care need, second only to prescription meds in the frequency of need [21]. Many of these children did not receive all of the care they needed, particularly those who were poor, uninsured, or had insurance lapses, and those with greater disability needs. Children with a personal doctor or nurse were significantly less likely to have unmet dental needs.

The multivariable logistic regression analysis in this study showed adolescents without a PCMH had 14% greater odds of ED visits. The bivariate analysis (Table 2) supports this result by showing three of the five Medical Home components (receiving family-centered care, not experiencing difficulty getting referrals, and receiving care coordination) were positively associated with fewer ED visits. This suggests that parents find it difficult to get the general and dental care they need for their adolescent with a DD if they lack the support some components of a PCMH offers. For example, within the bivariate analysis, adolescents who did not need a referral (87% reported No ED visit), compared to adolescents for whom it was not possible to get a referral (only 55% reported No ED visit). Likewise, for adolescents who did not need care coordination (47%), 90% had no ED visit, compared to families who did not receive the care coordination they needed (only 72% had no ED visit). Coordinating patient care is a standard practice within the Medical Home program [11]. While not able to be tested within this sample, children with a personal doctor or nurse are significantly less likely to have unmet dental needs [21].

There is little literature describing the Medical Home concept in dentistry, particularly for adolescents with DD. Care coordination was a featured part of the Alameda County Dental Service Utilization program [22] and programs designed to improve the successful transition of adolescents to adult care systems [23, 24]. Turchi et al. [25] reported that 68% of families with CSHCN who had some care coordination, encountered fewer problems with getting referrals for specialty care; were more likely to have family-centered care; felt a partnership with their professionals and satisfaction with services. They also reported fewer lost days of school and ED visits. Those who received no help with care coordination were more likely to be uninsured or on public insurance and had greater ED use. The guidelines produced by the American Association of Pediatric Dentistry Council on Clinical Affairs [26] stated that dental care comprehensive assessment of oral conditions should be made with referrals to dental specialists when care cannot be provided within the dental home. Implementation of such guidelines could help improve adolescents' oral health with DDs and reduce the family reliance on the ED.

This study had several strengths, including using a large national survey in which parents systematically gave input on the adolescent's oral and physical health, environment and access to healthcare. This enabled analysis of a broad set of characteristics. There are also limitations to the study, most apparent that it is cross-sectional, limiting any conclusions on causality. Further, information, such as the exact type of insurance, is not available and must be interpolated by the characterization provided. It is clear that not all insurance systems are identical. Parental reports *via* a survey do not give precise information regarding an adolescent's developmental disability and/or oral condition. Prospective studies involving methods to create a better definition of disability and oral condition status would help validate the results. Our study findings can be generalized only to non-institutionalized adolescents aged 10–17 years in the U.S. Utilization of ED visits for oral health-related issues are also observed internationally among children and adolescents [27, 28]. Implementing Medical Home principles within different country health systems and appropriate referrals to dentists may prevent dental-related ED visits among children and adolescents. Some subgroups in our sample seem over-represented, possibly due to non-response bias. However, no substantial evidence of non-response bias was reported by the Office of Management and Budget Standards after applying study weights in the 2016, 2107, 2018, and 2019 NSCH surveys. In addition, the weighted analysis of the data produced nationally representative data.

Bivariate analysis of which adolescents in this national sample were reported to have a Medical Home demonstrated that adolescents with DDs might not be receiving the support they need within the health care system (Table 4). Among this nationwide sample, a higher proportion of families had adolescents without DDs, private insurance, income higher than 400% FPL, and parents with a college degree or higher. Thus, a higher proportion of the very young people most in need of support in managing the health care system lacked the assistance of a Medical Home. Integrating the dental and medical health systems and incorporating the concepts of a Medical Home could be the first step to improving overall care for adolescents with disabilities.

## Implications

This cross-sectional analysis of adolescents investigated the number of ED visits using the components Andersen Behavioral Model. Having developmental disabilities and oral complications was associated with having a higher number of ED visits. Adolescents lacking the support of a Medical Home had a higher frequency of ED visits but Medical Home status was largely found among privileged adolescents.

## Data availability statement

The original contributions presented in the study are included in the article/supplementary materials, further inquiries can be directed to the corresponding author.

## Ethics statement

The studies involving human participants were reviewed and approved by the UCLA Office of the Human Research Protection Program determined that this secondary data analysis study did not meet the definition of human subjects research (IRB#21-00167). Written informed consent for participation was not required for this study in accordance with the national legislation and the institutional requirements.

## Author contributions

KA was involved in conceptualizing the project, selecting the appropriate variables, overseeing the data analyses, interpreting the data, devising the tables, and writing the manuscript. VB was involved in selecting the appropriate variables, overseeing the data analyses, interpreting the data, creating tables, and reviewing the manuscript. CW was involved in data analysis and management, interpretation, and manuscript review. All authors revised the manuscript critically for important intellectual content, reviewed the manuscript, and approved the final version.

## Funding

This project was supported by the Health Resources and Services Administration (HRSA) of the U.S. Department of Health and Human Services (HHS) under [R41MC42491] the Autism Secondary Data Analysis Research (SDAR) Program.

## Conflict of interest

The authors declare that the research was conducted in the absence of any commercial or financial relationships that could be construed as a potential conflict of interest.

## Publisher's note

All claims expressed in this article are solely those of the authors and do not necessarily represent those of their affiliated organizations, or those of the publisher, the editors and the reviewers. Any product that may be evaluated in this article, or claim that may be made by its manufacturer, is not guaranteed or endorsed by the publisher.

## Author disclaimer

The information, content, and/or conclusions are those of the authors and should not be construed as the official position or policy of, nor should any endorsements be inferred by HRSA, HHS, or the U.S. Government.
